# Histological and Immunohistochemical Insights into Disc Perforation in the Temporomandibular Joint: A Case Report

**DOI:** 10.3390/jfmk10020107

**Published:** 2025-03-27

**Authors:** Josè Freni, Antonio Centofanti, Fabiana Nicita, Davide Labellarte, Giovanna Vermiglio, Michele Runci Anastasi

**Affiliations:** 1 Department of Biomedical, Dental Sciences and Morphofunctional Imaging, University of Messina, 98122 Messina, Italy; jose.freni@unime.it (J.F.); antonio.centofanti@unime.it (A.C.); davidelabellarte99@gmail.com (D.L.); gvermiglio1@unime.it (G.V.); 2 Department of Maxillo-Facial Surgery, University of Sapienza, 00161 Roma, Italy; michele.runci@gmail.com

**Keywords:** temporomandibular joint disorders, anterior disc displacement, disc perforation, biomechanical stress, ECM degeneration

## Abstract

**Background/Objectives**: Anterior disc displacement without reduction (ADDwoR) is a temporomandibular joint (TMJ) disorder characterized by progressive dysfunction and potential complications. Persistent displacement leads to abnormal mechanical stress, predisposing the TMJ disc to structural degeneration, including perforation. This case report aimed to examine the histological and immunofluorescence characteristics of perforated disc tissue to elucidate the mechanisms contributing to its pathology. **Methods**: A 50-year-old patient with bilateral ADDwoR and disc perforation underwent functional arthroplasty. Tissue samples from the perforated disc were histologically analyzed using hematoxylin–eosin and Azan Mallory staining. Immunofluorescence was performed to assess the expression of collagen type I, fibrillin-1, matrix metalloproteinases (MMPs)-3 and -9, and cluster of differentiation 68 (CD68). **Results**: Histological analysis revealed disorganized collagen fibres and fibro-chondrocyte cell predominance in the perilesional zone, accompanied by vascular proliferation. Adjacent tissue to perforation exhibited normal fibrous organization. Immunofluorescence showed reduced collagen type I and fibrillin-1 patterns in the perilesional area, indicating an alteration in the fibrillar component of the extracellular matrix (ECM). Increased expression of MMP-3 and MMP-9, as well as elevated numbers of CD68-positive macrophages, suggested active ECM degradation and inflammation localized to the perforation site. **Conclusions**: This case report underscores the critical role of biomechanical stress and inflammation in disc perforation. Decreased ECM integrity, driven by altered collagen and fibrillin composition, as well as heightened MMP activity, compromises the disc’s capacity to absorb and distribute mechanical loads. These findings advance our understanding of TMJ pathophysiology, emphasizing the need for therapeutic approaches that target both biomechanical stabilization and inflammation.

## 1. Introduction

Temporomandibular joint (TMJ) disorders represent a widespread and complex clinical challenge in maxillofacial and dental medicine, encompassing a broad spectrum of pathological conditions [1,2,3,4,5]. Among these, anterior disc displacement without reduction (ADDwoR) stands out due to its potential to progress into chronic joint dysfunction, painful jaw locking, and progressive degenerative changes [6,7].

The TMJ disc, primarily composed of fibrocartilage and positioned between the condylar head and the glenoid fossa of the temporal bone, plays a critical role in ensuring smooth articulation and absorbing mechanical stress during jaw movements [8,9]. When the disc remains anterior to the condyle during mouth opening, as seen in ADDwoR, patients experience not only limited motion, but also ontological symptoms such as tinnitus, which may further deteriorate over time [10,11,12,13]. The etiology of ADDwoR is complex and multifactorial, involving several factors that increase strain on the TMJ structures, such as mechanical stresses, trauma, and parafunctional habits [14,15,16,17]. Persistent anterior displacement alters the joint’s biomechanics, resulting in increased compressive and shear forces that predispose specific regions of the disc to thinning and eventual perforation [18,19,20,21], particularly in the posterior area [22]. Once perforation occurs, the disc loses its capacity to effectively separate the joint surfaces, leading to joint inflammation, bone degeneration, and the onset of osteoarthritic changes [23,24].

Although disc perforations have been reported in advanced TMJ disorders and osteoarthrosis [25,26,27], their precise etiopathogenesis remains unclear, with prevalence estimates ranging widely from 6% to 42% [27,28,29]. While magnetic resonance imaging (MRI) is considered the gold standard for evaluating TMJ soft tissue [30,31,32], its ability to detect small perforations is limited by overlapping signal intensities [22,33,34], often necessitating direct visualization techniques, such as arthroscopy, for definitive diagnosis [23,27,35]. Additionally, the management of TMJ disc perforation depends on the extent of joint damage and the severity of the symptoms. Conservative treatments, such as splint therapy and pharmacologic interventions, aim to alleviate pain and inflammation, but are often inadequate once perforation has occurred [36]. In such cases, arthroscopic surgery is necessary to stabilize the joint and restore function [37,38]. Early diagnosis and intervention heavily influence the long-term prognosis of TMJ disc perforation [39].

Given these challenges, the histological examination of perforated TMJ discs is essential for unravelling the underlying mechanisms of disc degeneration. By analyzing these tissue-level changes, histology provides unique insights into the processes driving structural degeneration, helping to identify the factors that contribute to perforation and whose knowledge is currently very limited. In this context, we present a case of a patient with bilateral ADDwoR complicated by disc perforation, where detailed histological and immunofluorescence evaluations were performed to examine the distribution of disc tissue components and help understand the possible scenarios contributing to the pathology of TMJ disc perforation.

## 2. Materials and Methods

### 2.1. Case Presentation

A 50-year-old patient presented to the Department of Maxillofacial Surgery at Sapienza Hospital in Rome with the inability to close his mouth and return his TMJ to position. The patient’s medical history spanned 10 years of algic complaints, including headaches, neck pain, cervicobrachial pain, vertigo, and tinnitus. Approximately one year after the onset of his initial symptoms, he began experiencing episodes of joint clicking and limited jaw function.

Initially, he consulted a physiotherapist, which partially alleviated his muscle tension symptoms; however, the clicking persisted, along with the development of pre-auricular pain. Subsequently, he was referred to a dentist and prescribed a night guard, followed by a daytime device due to lack of benefit. Over the next three years, the joint clicking gradually disappeared, but his local TMJ pain, muscle pain, and otologic symptoms worsened. The patient reported a sensation of “locking” in the joint and increased tinnitus and limitations in TMJ movement, which severely impacted his daily activities, including difficulties in mouth opening and chewing. He also had a history of TMJ dislocations, initially managed by self-intervention or dental clinic visits; however, the frequency and severity of these dislocations increased over time. The patient had no history of previous trauma, parafunctional habits, or systemic diseases. In an emergency, a nonsurgical attempt at reducing the dislocated joint was manually performed.

On clinical examination, palpation revealed high-intensity pain in the area around the TMJ and significant muscle tension in the masticatory muscles. The pain assessment was measured using a visual analogue scale (VAS) ranging from 0 (“no pain”) to 10 (“unimaginable pain”) [40], with the patient reporting a score of 9. Range of motion (ROM) assessments, performed according to the DRC/TMD guidelines [6] using a calibrated metal ruler, revealed a maximum mouth opening of 30 mm, lateral excursions of 6 mm on the right and 5 mm on the left, and a protrusive movement limited to 4 mm. The end feel test ruled out muscle limitation. No facial asymmetry was noted during clinical evaluation.

**Figure 1:**
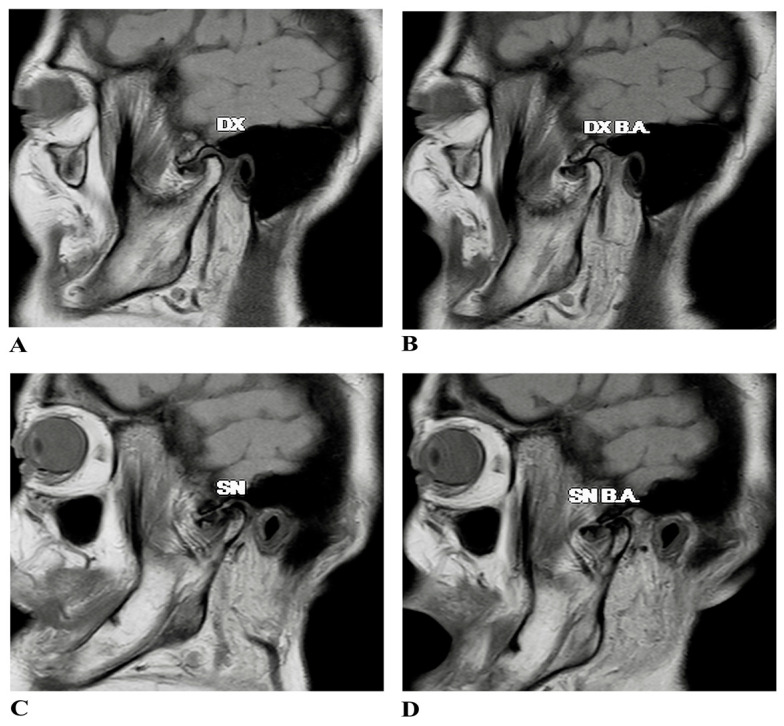
Proton density (PD)-weighted magnetic resonance imaging (MRI) of the right and left TMJs in closed and open mouth positions. (**A**–**D**) The MRIs of both TMJs show anterior disc dislocation without reduction. (**A**,**C**) In the closed position (maximum intercuspal), the intermediate zone of the disc is situated anterior to the condylar head, with the posterior part located anterior to the 11:30 position. (**B**,**D**) The intermediate zone maintains its anterior position relative to the condylar head in the open position.

Radiographic evaluations, including orthopantomography and computed tomography (CT), revealed osteoarthritic changes in both TMJs, with more advanced degeneration on the left side. Magnetic resonance imaging (MRI) further confirmed a bilateral ADDwoR, providing a detailed visualization of soft tissue structures and establishing a baseline for surgical intervention (Figure 1).

### 2.2. Surgical Treatment

**Figure 2:**
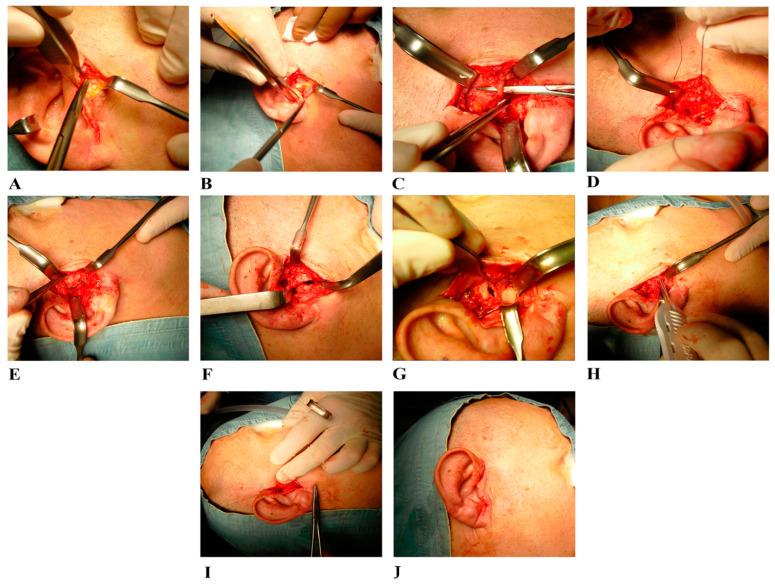
Representative surgical steps of the functional arthroplasty procedure performed on the left TMJ. (**A**) Pre-auricular retro-tragal incision exposing the superficial and deep temporalis fascia. (**B**) Detachment of the parotid gland from the tragus cartilage and TMJ capsule. (**C**) Identification of the superficial temporal artery and vein. (**D**) Ligation and interruption of the superficial temporal artery and vein. (**E**) Exposure of the joint capsule by blunt dissection and identification of the lateral ligament insertion on the condyle. (**F**) Incision of the lateral ligament to access the inferior compartment. (**G**) Recapture of the anteriorly displaced disc. (**H**) Stabilization of the disc position using anchor screw discopexy and lateral ligament. (**I**) Deep plane sutures. (**J**) Skin sutures.

The initial management plan involved conservative treatments, including the prescription of nonsteroidal anti-inflammatory drugs (NSAIDs) to manage pain and reduce inflammation. An occlusal splint was also recommended to relieve muscle tension and reduce TMJ loading while awaiting the planned surgery. Due to the increased severity of symptoms, functional arthroplasty (FA) [41] was performed in the left TMJ (Figure 2).

The patient underwent nasotracheal general anesthesia, allowing occlusion control throughout the procedure. Antibiotic prophylaxis with intravenous cephalosporin was administered one hour before surgery, and a trichotomy was performed on the hair anterior to the affected ear. The surgical field, including the ipsilateral temporal area and oral cavity, was thoroughly cleansed with chlorhexidine digluconate and 70% alcohol solution, and sterile drapes were applied. The ipsilateral temporal area was left uncovered to monitor facial nerve activity. The patient was positioned in a semi-seated position with the head turned to expose the affected TMJ. A pre-auricular retro-tragal incision was made to expose the superficial and deep temporalis fascia, avoiding the frontal branch of the facial nerve above the superficial fascia (Figure 2A). The parotid gland was carefully detached from the tragus cartilage and the TMJ capsule (Figure 2B). The condyle’s mobility was assessed with mandibular movement, and the superficial temporal artery and vein were identified (Figure 2C), tied off, and interrupted (Figure 2D). Then, the surgeon used blunt dissection to expose the joint capsule, placing the lateral ligament’s insertion on the condyle (Figure 2E). Additionally, the condition of the TMJ superior compartment was checked by diagnostic arthroscopy, which revealed the presence of synovitis. After performing arthrocentesis with continuous irrigation and lavage using a sterile solution, the lateral ligament was incised, allowing the disc to be moved upward and exposing the inferior TMJ compartment (Figure 2F). To reshape the top of the condylar head and improve the stability of the disc on the condyle, a condylar shaving was performed. An anteromedial disc perforation was identified and repaired. Perforated 3 × 2 mm disc tissue was collected, carefully managed to preserve integrity, and prepared for histological examination. Then, the disc was anatomically repositioned over the condylar head (Figure 2G), and the anchor screw discopexy with lateral ligament was used to stabilize the disc’s position (Figure 2H). Finally, the deepest plane sutures and then skin sutures were carried out (Figure 2I,J).

### 2.3. Samples Collection

The disc-perforated sample from surgery and the control autoptic sample were fixed in 4% paraformaldehyde within a 0.05 M phosphate-buffered saline (PBS) solution at 4 °C. They underwent dehydration using ethanol, clearing in xylene, and were embedded in Paraplast (SPI Supplies, West Chester, PA, USA) [42]. The blocks were sliced with a microtome (RM2125 RT, Leica Instruments, Nussloch, Germany), and 7 μm sections were then mounted onto glass-silanized slides.

### 2.4. Light Microscopy

The sections of perforated disc were cleared with xylene, rehydrated in ethanol, and stained with hematoxylin and eosin and the Azan Mallory trichromatic method, according to the manufacturer’s protocol (Bio-Optica Milano s.p.a, Milan, Italy) [43]. All samples were examined using a Nikon Ci-L light microscope (Nikon Instruments, Tokyo, Japan). Micrographs were captured with a Nikon Ds-Ri2 digital camera and saved as tagged image format files (TIFF) using Adobe Photoshop software CS5 12.1 [44].

The disc sections were reconstructed to visualize all four microscopic field areas: anterior, lateral, medial, and posterior. The perforation corresponded to the anteromedial area. We named the area near the perforation the “perilesional area” and the zone near the perilesional area the “healthy disc area”.

### 2.5. Immunofluorescence

Both perforated disc sections and control autoptic disc sections were treated as primarily described and placed on silanized glass slides. After deparaffination, the sections were thoroughly washed in phosphate-buffered saline (PBS) and preincubated with 1% bovine serum albumin (BSA) and 0.3% triton X-100 in PBS for 30 min at room temperature to block nonspecific binding sites and to permeabilize the membranes. Finally, the sections were incubated overnight at 4° with following primary monoclonal antibodies diluted in PBS-BSA: anti-collagen type I (Invitrogen, Thermo Fisher Scientific, Waltham, MA, USA), dilution range 1:100; anti-fibrillin 1 (Santa Cruz Biotechnology Inc., Santa Cruz, CA, USA), dilution range 1:100; anti-cluster differentiation 68 (CD68) (Ventana, Roche Diagnostic, Basel, Switzerland), dilution range 1:2.5; anti-matrix metallopeptidase-9 (MMP-9) (Abcam, Cambridge, UK), dilution range 1:100 and anti-matrix metallopeptidase-3 (MMP-3) (Abcam, Cambridge, UK), dilution range 1:100. The following primary antibodies: anti-CD68, anti-collagen type I, were identified with Texas-red-conjugated immunoglobulin (IgG) anti-mouse (Jackson ImmunoResearch Laboratories, Inc., West Grove, PA, USA) [45]; anti-fibrillin-1 was detected using fluorescein isothiocyanate (FITC)-conjugated immunoglobulin anti-mouse (IgG) (Jackson ImmunoResearch Laboratories, Inc., West Grove, PA, USA) [46]; anti-MMP9 and anti-MMP3 were detected using fluorescein isothiocyanate (FITC) immunoglobulin (IgG) anti-rabbit (Jackson ImmunoResearch Laboratories, Inc., West Grove, PA, USA), all at a dilution range of 1:100 [47]. The sections were analyzed, and images were acquired using an ECLIPSE Ni E200 MV fluorescence microscope (Nikon Instruments Inc., Melville, NY, USA). All images were digitized at a resolution of 8 bits, resulting in a 2048 × 2048-pixel array. Optical sections of fluorescence specimens were captured using a RED laser (wavelength = 596 nm) and a GREEN laser (wavelength = 485 nm). We set the contrast and brightness by inspecting the most intensely labelled pixels and selecting settings that revealed structural details while maintaining the highest pixel intensity (200). Each image was captured within 62 s to reduce photodegradation [48]. Digital images were cropped, and figure montages were prepared using Adobe Photoshop 8.0 (Adobe System, Palo Alto, CA, USA). An observer performed a qualitative analysis based on the colour intensity for all markers analyzed with the immunohistochemical method.

## 3. Results

### 3.1. Histological Evaluation

**Figure 3:**
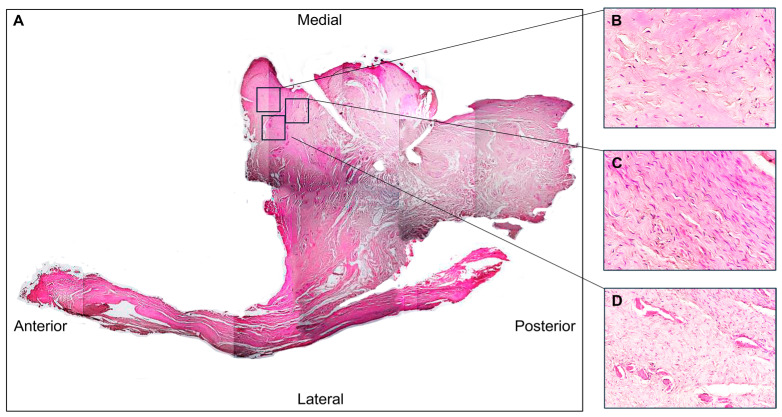
(**A**) Hematoxylin and eosin staining of the TMJ disc (4× magnification). (**B**) Disorganized collagen fibres are present in the perilesional area, along with numerous vessels (**D**) (magnification 40×). (**C**) The healthy disc area, adjacent to the perilesional area, shows thick bundles of collagen running in a parallel manner (magnification 40×).

The hematoxylin–eosin staining revealed a poorly organized dense connective tissue in which collagen fibres were randomly oriented, forming thin and scattered structures (Figure 3A). This morphological feature was especially evident in the perilesional area (Figure 3B), where disarranged fibres or a complete lack of aggregation were observed (Figure 3C). The healthy disc area (Figure 3C) showed the normal densely woven fibrous tissue, in which collagen fibres aligned parallel to form thick collagen bundles.

In addition, cells with an elongated shape typical of fibroblasts can be observed in the healthy disc area (Figure 3C); on the contrary, in the peilesional area, the cells exhibit an oval shape typical of fibrocyte (Figure 3B). In addition, the presence of numerous vessels was evident, particularly in the region adjacent to the perforation (Figure 3D).

**Figure 4:**
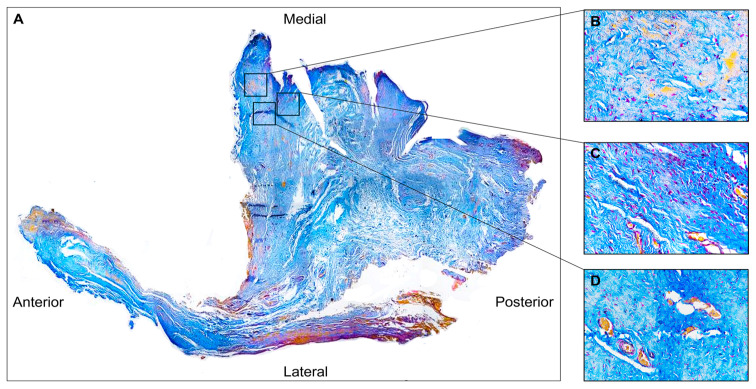
(**A**) Azan Mallory staining of TMJ disc (4× magnification). (**B**) Disorganized collagen fibres are present in the perilesional area indicated by reduced blue staining (40× magnification). (**C**) The adjacent normal disc area has parallel collagen bundles (40× magnification). (**D**) Numerous vessels are present in the perilesional region (40× magnification).

The Azan Mallory trichrome staining in the perilesional area highlighted fibre reduction and fragmentation, as evidenced by the weak blue colour staining (Figure 4B).

### 3.2. Immunofluorescence Evaluation

**Figure 5:**
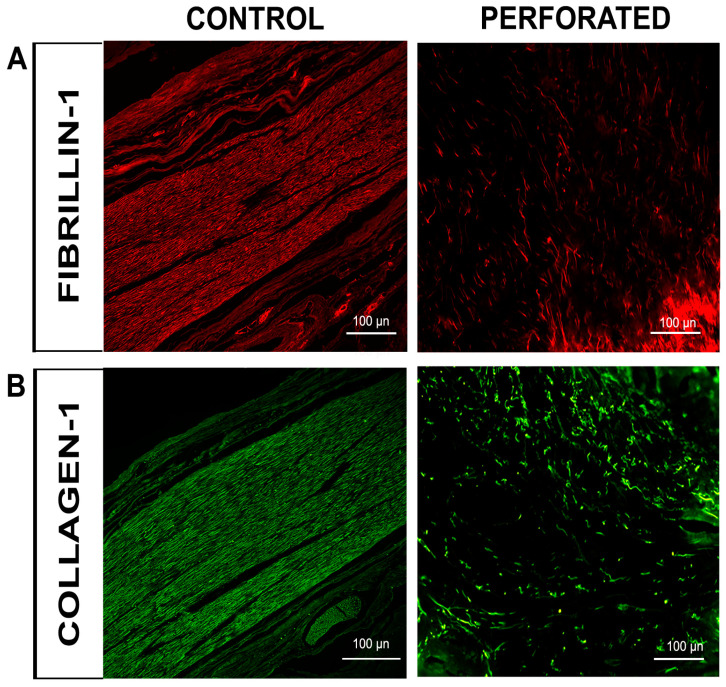
Compound panel of immunofluorescence single-localization reactions in perforated and control TMJ discs, using antibodies against fibrillin-1 ((**A**), green channel) collagen type I (**B**) in the red channel. The intensity of both fluorescence patterns is significantly reduced in the perilesional area of the perforated disc if compared to the control disc (magnification 20×).

The single-localization immunofluorescence reactions with anti-collagen type I and anti-fibrillin-1 antibodies revealed distinct staining patterns: fibrillin-1 was detected in the red channel (Figure 5A), while collagen type I was observed in the green channel (Figure 5B) for both normal and perforated TMJ disc samples. The fluorescence intensities of both collagen I and fibrillin-1 markers were significantly lower in the perilesional area compared to the control disc. Furthermore, in the perilesional zone, the fluorescence pattern for both markers also appeared discontinuous and structurally disorganized, with noticeable gaps between regions. In contrast, the control displayed a uniform and concentrated signal (Figure 5A,B).

**Figure 6:**
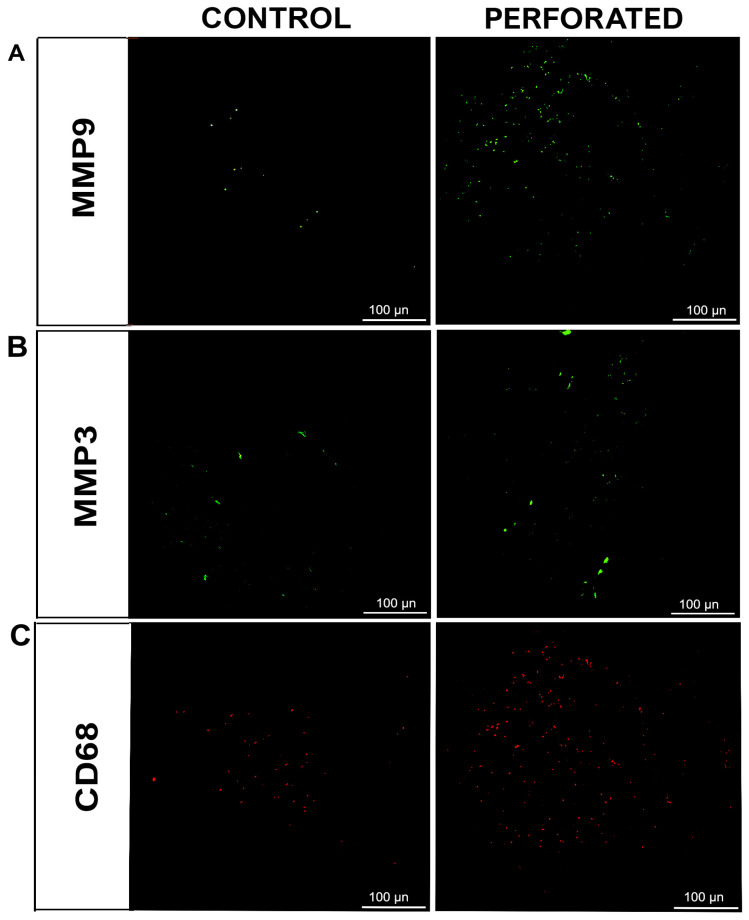
Compound panel of immunofluorescence single-localization reactions in perforated and normal TMJ disc sections, using antibodies against MMP-9 ((**A**), **green channel**) and MMP-3 ((**B**), **green channel**) and CD68 ((**C**), red channel). The fluorescence staining patterns for all markers show an increase in the perilesional area of the perforated disc if compared to the control (magnification 20×).

Furthermore, additional single-immunofluorescence analysis revealed MMP-9 in the green channel (Figure 6A), MMP-3 also in the green channel (Figure 6B), and CD68 in the red channel (Figure 6C) in both normal and perforated disc. Notably, the CD68 marker, indicative of macrophage-like cells, displayed significantly enhanced fluorescence in the perilesional zone of the perforated disc compared to the normal disc tissue, as well as the fluorescence pattern of the MMP-9 marker, indicative of proteolytic enzyme (Figure 6A,C).

In contrast, the signal for MMP-3, indicative of a proteolytic enzyme, was slightly weaker than the other two markers in the perforated disc sample but remained considerably higher than in the healthy disc sample (Figure 6B).

## 4. Discussion

This case report presents a pioneering histological investigation of a perforated human TMJ disc, providing valuable insights into the microstructural changes underlying temporomandibular disorders (TMDs). Although TMJ disc perforation has been extensively documented through imaging and clinical studies [47,48,49], this research highlights the distinct benefits of microscopic examination in elucidating the cellular and tissue changes that lead to joint dysfunction. This approach enhances our understanding of the pathophysiological mechanisms driving TMJ disc perforation and its implications for joint integrity and function.

The investigation into collagen type I, fibrillin-1, MMP-3, and MMP-9 in TMJ disc perforation is based on their critical roles in maintaining disc structural stability and extracellular matrix (ECM) integrity. Collagen type I, the main fibrillar protein within the TMJ disc, provides tensile strength and resistance to mechanical deformation [49,50]. Its organization into stress-aligned bundles enhances the disc’s ability to withstand compressive and shear forces during activities such as chewing and jaw movements [51]. Fibrillin-1, another key ECM component, contributes to the disc’s elasticity and resilience by forming microfibrils that interact with collagen, enabling the disc to recover its shape after cyclic loading [52,53]. These proteins form an integrated structural framework that is essential for shock absorption and force distribution. Conversely, MMPs, like MMP-3 and MMP-9, were examined for their known roles in ECM degradation, as their enzymatic activity targets collagen, elastin, proteoglycans, and other ECM components [54,55,56]. The upregulation of these MMPs in pathological conditions often reflects an imbalance between tissue repair and degradation, leading to structural breakdown [54].

Histological and immunofluorescence analyses revealed a complex interplay between degeneration, inflammation, and ECM remodelling associated with TMJ disc perforation. Hematoxylin–eosin staining showed disorganized and fragmented collagen fibres in the perilesional area, consistent with previous studies that reported significant collagen degradation in perforated discs [57,58]. Collagen fibres were sparsely aggregated and exhibited an irregular arrangement. Azan Mallory staining highlighted the degree of collagen fragmentation and the disruption of matrix continuity. These alterations suggest the hypothesis of a compromised ability of the disc to withstand mechanical stress, further predisposing it to structural failure. Furthermore, a notable reduction in fibroblast-like cells in the perilesional zone was observed, suggesting a diminished capacity for collagen synthesis and remodelling. Immunofluorescence evaluation confirmed the histological findings, showing a reduction in collagen type I and fibrillin-1 patterns in the perilesional zone, indicating compromised ECM integrity and impaired structural support. The reduced signal intensity in these key matrix components compared with the control sample underscores the localized tissue degradation at the lesion site.

Microscopic analysis of TMJ discs with perforations has highlighted significant fibrocartilage degeneration. Studies have reported a loss of collagen integrity and increased inflammatory cell infiltration as hallmarks of the response to TMJ disc injuries [57,58]. The presence of MMPs, notably MMP-7 and MMP-9, has been documented in the context of TMJ disc degeneration, indicating that these enzymes play a role in the degradation of the ECM fibrillar components following disc perforation [57,59]. Our immunofluorescence findings of increased MMP-3 and MMP-9 expression in the perilesional area align with the existing literature, suggesting that these enzymes could play a role in the progressive degeneration of the disc and subsequent loss of structural integrity. Additionally, the upregulation of inflammatory markers in the ruptured area, such as CD68-positive macrophage-like cells, indicates an active response to tissue damage. The inflammatory response associated with TMJ disc perforation has been reported by Luo et al. [60], noting a rise in pro-inflammatory cytokine activity that could worsen tissue damage and trigger further degeneration. The presence of numerous vessels, predominantly in the perilesional zone, aligns with studies of increased angiogenesis in response to TMJ disc perforation. This observed vascular growth is likely driven by elevated levels of the vascular endothelial growth factor (VEGF), as reported by Feng et al. [58], which acts as a response to inflammatory conditions. Such vascular proliferation could facilitate the infiltration of inflammatory cells and contribute to the local degenerative process.

In contrast, the healthy disc area of the perforated disc exhibited densely woven, parallel collagen fibres, consistent with its role in maintaining tensile strength and normal biomechanics [61,62,63]. This clear distinction between the healthy disc and perilesional areas within the same sample highlights the localized nature of tissue degeneration in TMJ disc perforation, underscoring the interplay between altered mechanical forces and structural changes.

These observations offer a new perspective on the pathophysiology of TMJ disc perforation, which has remained unclear due to the limited availability of histological studies. Notably, similar patterns of collagen degradation, fibrotic changes, and inflammatory response have been observed in other articular disc pathologies, suggesting that common mechanisms of disc degeneration might exist across different joint structures. By documenting the cellular and molecular alterations in a perforated TMJ disc, our study lays the groundwork for future investigations into potential therapeutic targets for disc-related disorders. However, being a case report based on a single patient, the findings may not be widely generalizable. We recommend that future studies utilize larger patient cohorts and adopt longitudinal designs to capture the progression of tissue changes better. Prospects also include further exploration of the molecular pathways involved in ECM degradation and inflammation within TMJ disc pathology. A deeper understanding of these tissue alterations could guide the development of more effective treatments, such as biologic therapies aimed at restoring disc integrity or modulating the inflammatory response.

Based on our histological findings, we recommend that clinical practice incorporate early and comprehensive diagnostic evaluations for TMJ disorders. Early intervention should be considered when signs of disc degeneration or perforation are identified. While conservative treatments remain the first line of management, surgical repair is necessary in cases of confirmed perforation to restore joint stability and function. Overall, integrating advanced diagnostic techniques with a personalized treatment approach may enhance patient care by preventing progression to severe joint dysfunction and optimizing functional recovery.

## 5. Conclusions

This case report provides novel histological insights into TMJ disc perforation. The disorganization of collagen type I and fibrillin-1, along with the upregulation of MMP-3 and MMP-9, underscores the critical role of extracellular matrix degradation in compromising disc integrity. These findings emphasize the interplay between mechanical stress, inflammation, and ECM remodelling in TMJ disc perforation, laying the groundwork for targeted therapeutic strategies and demonstrating the value of histological analysis in understanding the processes underlying TMJ disorders.

## Data Availability

The data can be obtained upon request from the corresponding author. The data are not publicly accessible owing to privacy concerns.
